# Veteran Monitoring Initiative for Noninvasive Physiology and Depression (V-MIND) Exploring Physical Activity and Mental Health in UK Veterans: Protocol for an Observational Digital Phenotyping Study

**DOI:** 10.2196/73060

**Published:** 2026-04-29

**Authors:** Danielle Dryden, Daniel Leightley, Natasha Biscoe, Dominic Murphy

**Affiliations:** 1 Combat Stress Centre for Applied Military Health Research Surrey United Kingdom; 2 Department of Population Health Sciences, Faculty of Life Sciences & Medicine, King’s College London London United Kingdom; 3 University of Oxford Oxford United Kingdom; 4 Kings Centre for Military Health Research, King’s College London London United Kingdom

**Keywords:** common mental disorders, military, physical activity, veterans, wearable technology

## Abstract

**Background:**

Veterans face an increased risk of common mental disorders when compared to civilian groups. However, veteran disengagement from treatment is a concern among health care providers, resulting in a need to explore novel ways of managing veteran mental health. Wearable devices, such as fitness trackers and smartwatches, have been explored for their potential to assess, monitor, and predict mental health outcomes in the general population. Such devices provide continuous data on metrics including physical activity, heart rate, sleep quality, and stress levels, offering a comprehensive view of the lifestyle and physiological factors influencing mental health.

**Objective:**

This study aims to explore the feasibility of using wearable technology as a data collection and potential health monitoring tool among UK veterans. It also aims to explore the associations between mental health, physical activity, and functioning factors among UK veterans.

**Methods:**

This is an observational feasibility study measuring mental health via validated questionnaires completed at baseline (T0), day 28 (T1), day 56 (T2), and day 84 (T3), and physiological metrics measured continuously via wrist-worn fitness trackers (Garmin vívosmart-5 watches) over 3 months (84 days). UK veterans will be recruited through convenience sampling methods. Statistical analysis will be exploratory, and machine learning models will be trained to detect changes in mental health and well-being outcomes.

**Results:**

Data collection was conducted between February 2025 and October 2025, and data analysis is scheduled to begin in January 2026.

**Conclusions:**

This study will provide information on the feasibility of using wearable technology devices within a UK veteran population and may inform potential future interventions seeking to integrate wearable-derived data alongside the management of common mental disorders in veterans experiencing mental health difficulties. Findings would also enhance understanding of the relationship between mental health and physiological factors (eg, physical activity and sleep) in UK veterans.

**International Registered Report Identifier (IRRID):**

PRR1-10.2196/73060

## Introduction

Veterans face an increased risk of common mental disorders (CMDs), with depression rates estimated to be approximately double that of the general population (40%) [1]. In the United Kingdom, a veteran is defined as an individual who has completed a minimum of 1 day’s paid employment in the Armed Forces [2]. There are estimated to be 2.03 million Armed Forces veterans in Great Britain [3,4], with additional numbers in Northern Ireland estimated between 40,000 and 60,000 [5].

While most veterans have positive in-service experiences, for some, their time in the military may lead to adverse mental health outcomes. Unique military occupational demands mean veterans may encounter stressors not experienced by civilian populations, such as combat exposure [6], which has been associated with an increased risk of experiencing adverse mental health outcomes, including posttraumatic stress disorder (PTSD) and depression [7].

The impact of depression, for instance, extends beyond mental health, increasing the risk of physical health problems such as type 2 diabetes and cardiovascular disease [8], as well as mental health comorbidities including PTSD, anger, anxiety, and alcohol misuse [9]. Further, the economic cost of depression in the United Kingdom is substantial, estimated as 5% of the country’s GDP [10]. In a sample of 693 veterans identified from clinical databases and accessing secondary mental health services in the United Kingdom, the rate of depression was identified as 22.9% [11]. Additionally, in a sample of UK veterans experiencing mental health difficulties who were deployed during the Iraq and Afghanistan conflicts, CMDs were identified as the most prevalent mental health problem (27.8%), followed by probable PTSD (9.4%) and alcohol misuse (8.4%) [12]. However, veteran disengagement from available treatment is a concern among mental health service providers, with many veterans either not seeking or discontinuing treatment early [13]. The need to explore novel ways of managing mental health difficulties in this population is clear.

Wearable devices, such as fitness trackers and smartwatches, have been explored for their potential to assess, monitor, and predict depression in patients [14,15]. Such devices can provide continuous data on an individual’s physical activity, heart rate, sleep quality, and stress levels, offering a comprehensive view of the lifestyle and physiological factors potentially influencing mental health. Wrist-worn devices are nondistracting and easy to administer to participants. They have been proposed as an effective way of collecting data in real time and have been successfully used to explore the association between depressive symptoms and wearable-derived features such as heart rate variability (HRV) [16], sleep, and physical activity in civilian samples [17]. Research investigating the relationship between mental health and features measurable via wearable technology devices is increasing, and health care providers are becoming increasingly focused on the potential use of wearable technology as a tool for health monitoring in the general population [18].

Military health research specifically has demonstrated the feasibility and acceptability of technology-driven methods in the management of veteran health, which offer a promising avenue for future interventions. For example, the successful use of a mobile app designed to manage harmful drinking behaviors demonstrates the potential of such interventions [19]. Real-time symptom monitoring and novel diagnostic and treatment approaches, such as behavioral activation through physical activity, could be key in the management of veteran mental health; however, there is limited research currently available within veteran groups exploring this [18]. As such, this study will use wearable technology (Garmin watches, vívosmart-5 model) to observe the relationship between mental health, physical activity, and functioning factors such as sleep among a UK veteran sample. Findings from this study may inform potential future interventions seeking to integrate wearable device data alongside the management of CMDs in veterans experiencing mental health difficulties.

This study aims to explore the associations between CMDs, including symptoms of anxiety and depression measured monthly using the 12-item General Health Questionnaire (GHQ-12), and indicators of physical activity recorded by wrist-worn fitness trackers among UK veterans. Garmin-derived metrics include steps, active minutes per day, HRV, stress levels, and overall activity patterns. In addition, the study seeks to examine the relationship between mental health and broader functioning factors, such as somatic symptoms, loneliness, and sleep, in relation to physical activation metrics (steps, active minutes per day, HRV, stress, and activity) obtained from fitness trackers. The primary outcome of interest is the change in GHQ-12 score from baseline (T0) to day 84 (T3) associated with the change in physical activity over the 3-month study period. The study also aims to assess the feasibility of using fitness trackers to enhance the prediction and management of CMDs among UK veteran populations, to inform the development of a potentially larger-scale initiative. Feasibility will be assessed through recruitment rate, retention rate, and data completeness of both watch-derived data and monthly questionnaire responses. By integrating objective physiological data with self-reported mental health measures, this work ultimately aims to support improved monitoring and intervention strategies for CMDs within the veteran population.

## Methods

### Ethical Considerations

#### Ethics Approval

The study protocol and all related research material (informed consent forms, participant information sheet, recruitment materials, and assessments) were reviewed and approved by the King’s College London Research Ethics Committee (reference number MRA-24/25-45701) via a minimal risk self-registration process, in line with King’s College Research Ethics Committee minimal risk guiding principles.

#### Privacy and Confidentiality

**Figure 1:**
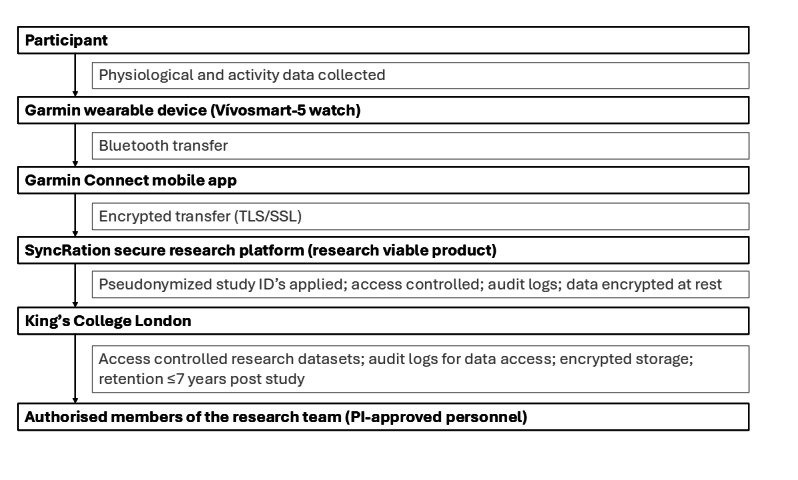
Data flow of wearable-derived data. PI: principal investigator; SSL: Secure Sockets Layer; TLS: Transport Layer Security.

The study will collect limited personally identifiable information (name, contact details, service number verification, address, and study entry date) solely for participant communication and study administration. Identifiable data will be held securely by King’s College London and Combat Stress, stored separately from the research dataset, with access restricted to authorized members of the research team as approved by the principal investigator. Personally identifiable information will be held for a period of up to 1 year after the study has been completed. All data processing will comply with the General Data Protection Regulation (GDPR; specifically, the GDPR 2018 Act of Parliament) and institutional data–governance policies. Wearable-derived data will be transferred from the Garmin Connect app to SyncRation, the approved secure research platform used at King’s College London, via encrypted protocols (Transport Layer Security/Secure Sockets Layer) and stored on access-controlled servers with encryption at rest. Systems used by the research team maintain audit logs of data access and changes, and operate within protected, segmented network environments to reduce security risk. Identifiable information is stored separately from research datasets, with linkage performed through pseudonymized study identifiers. Garmin does not have access to any identifiable research data. Nonidentifiable information will be held for a period of up to 7 years after the study has been completed (Figure 1).

#### Informed Consent Procedures

If the participant agrees to take part, informed consent will be obtained at the end of the online screening questionnaire before the collection of any data. Participants will be informed that they can withdraw from the study at any time. All data, including those from withdrawn participants, except for those who request that their data be deleted/not used, will be included in the final analysis.

#### Compensation

Participants will be gifted a £10 (US $13.40) Amazon voucher upon completion of the study. They will also be gifted the Garmin vívosmart-5 watch used to collect study data free of charge; the watch is valued at £129.99 (US $174.19).

### Eligibility Criteria

Participants will be veterans of the UK Armed Forces who meet caseness for CMDs, including symptoms of anxiety and depression, defined by a cutoff score of ≥12 on the GHQ-12. A brief online survey will screen for study inclusion criteria and obtain informed consent from participants (Textbox 1). Eligible participants will receive a phone call from a member of the research team to confirm their delivery address for the Garmin watch, their status as a veteran via their service number, and their service history (when they served and when they left service).

**Participant eligibility criteria.**

**Inclusion criteria**

- UK military veterans aged ≥18 years. In the United Kingdom, individuals are defined as veterans if they have completed a minimum of 1 day of paid employment in the UK Armed Forces [20]
- Self-reported experiences of common mental disorders, including symptoms of anxiety or depression, caseness identified by a score of ≥12 on the 12-item General Health Questionnaire [21]
- Fluent (speaking and reading) in English
- Use an iOS or Android device released within the previous 5 years
- Access to a USB-C compatible charging port

**Exclusion criteria**

- Living outside of the United Kingdom
- Currently serving
- Unwilling and/or unable to provide informed consent

### Study Design

An observational feasibility study in which 50 UK veterans who self-report experiencing CMDs, including symptoms of anxiety and depression, will be recruited through convenience sampling methods. The target population for this study is not clinical, and there are no comparators. Participants will wear a Garmin watch, delivered to their home address, for 84 days, and complete baseline measures plus 3 monthly questionnaires about their mental health and well-being, administered on Qualtrics survey software (Qualtrics Labs, Inc). Participants will wear the devices in their everyday environment and will be sent automated email reminders to complete the monthly surveys.

**Figure 2:**
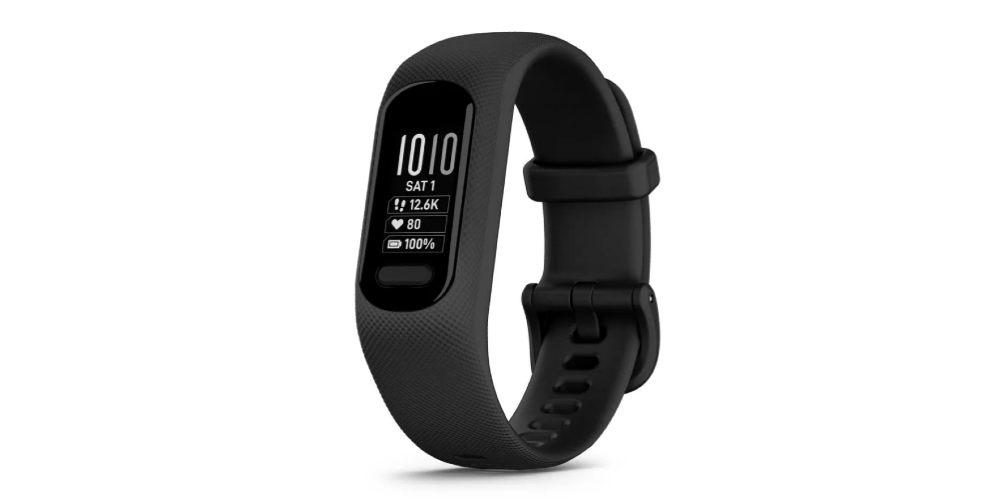
Sample devices (Black).

Participant self-reported mental health and well-being data will be collected via monthly online questionnaires (a summary of the measures used is detailed in Table 1), and behavioral and physiological indices will be measured and collected using Garmin watches (vívosmart-5 model; Figure 2). Data will be collected via the watches on the following outcomes continuously (24/7): activity level (number of steps, active minutes, and self-reported activity), HRV, overall body battery, sleep (sleep quality, sleep score, and hours of sleep), and stress.

**Table 1:** Mental health and well-being measures.

Mental health and well-being measures	Description of measure	Cutoff
Alcohol Use Disorders Identification Test for Consumption [22]	3-item measure of heavy drinking and/or active alcohol dependence. Assesses frequency and quantity of alcohol consumption, and frequency of high-intensity drinking episodes (≥6 units for women, ≥8 for men).	Scores range from 0 to 12. ≥5 indicates a positive screen. Categories: low risk (0-4), increasing risk (5-7), higher risk (8-10), and possible dependence (11-12).
12-item General Health Questionnaire [21]	Measure of common mental disorders, focusing on non-psychotic symptoms such as depression and anxiety.	Scores range from 0 to 36 (Likert scale). Caseness is defined as ≥12.
Generalized Anxiety Disorder-7 [23]	Screens for and assesses the severity of generalized anxiety disorder.	Scores range from 0 to 21. Categories: minimal (0-4), mild (5-9), moderate (10-14), and severe (15-21).
Patient Health Questionnaire-2 [24]	Brief screening tool for depression assessing frequency of low mood and anhedonia over the past 2 weeks.	Scores range from 0 to 6. ≥3 indicates probable depression.
Patient Health Questionnaire-15 [25]	Measures the severity of somatic (physical) symptoms such as pain and fatigue.	Scores range from 0 to 30. Categories: minimal (0-4), low (5-9), medium (10-14), and high (15-30).
Posttraumatic Stress Disorder Checklist-5 [26]	Assesses presence and severity of PTSD^a^ symptoms aligned with DSM-5 criteria; used for screening and monitoring over time.	Scores range from 0 to 80. ≥31 indicates provisional PTSD diagnosis.
Pittsburgh Sleep Quality Index [27]	Assesses sleep quality and disturbances over a 1-month period.	Scores range from 0 to 21. ≥5 indicates significant sleep disturbance.
UCLA^b^ Loneliness Scale-3 [28]	Brief 3-item measure of perceived loneliness.	Scores range from 3 to 9. Categories: not lonely (3-5) and lonely (6-9).
Warwick-Edinburgh Mental Well-being Scale-7 [29]	Measures positive mental well-being, including aspects such as personal growth, autonomy, and relationships; shortened 7-item version.	Scores range from 7 to 35. Categorized using ±1 SD as low, normal, or high well-being.
Dimensions of Anger Reactions-5 [30]	Brief standardized measure of trait anger; sensitive screening tool for anger-related difficulties.	Scores range from 5 to 25. ≥12 indicates problematic anger.
Insomnia Severity Index [31]	Brief screening tool measuring the severity of insomnia symptoms.	Scores range from 0 to 28. Categories: none (0-7), subthreshold (8-14), moderate (15-21), and severe (22-28).
International Physical Activity Questionnaire Short Form [32]	Assesses physical activity across vigorous, moderate, walking, and sitting domains over the past 7 days.	Produces MET^c^-min/week. Categories: low, moderate, high physical activity.
Perceived Stress Scale-10 [33]	Measures perceived stress based on feelings of unpredictability, uncontrollability, and overload over the past month.	Scores range from 0 to 40. Categories: low (0-13), moderate (14-26), and high (27-40).

^a^PTSD: posttraumatic stress disorder.

^b^UCLA: University of California, Los Angeles.

^c^MET: metabolic equivalent of task.

A pilot study, with 5 participants, will be conducted prior to commencing the study. The pilot study will be conducted for the purpose of testing the study design and study schedule. The pilot study sample is made up of experts working in the field of military mental health who have experience working with the veteran population. Feedback from the pilot study will be used solely to inform the main study design and study schedule; any alterations identified from the pilot study will be made before the main study commences. Data collected from the pilot study will not be used in the analysis of results from the main cohort.

### Study Schedule

A recruitment advert will include a link to the registration survey where participants can register their interest in taking part in the study. This survey will collect participants’ consent to be contacted about the study, contact details, and a copy of the participant information sheet will be included, and informed consent to participate in the study will be collected. Prospective participants will confirm they meet the inclusion and exclusion criteria on this survey. Those who do not meet the inclusion criteria for the study will be informed via email. Eligible participants will be invited to attend an online verification call conducted with a member of the research team. During this call, participants will be given the opportunity to ask any questions they may have about the study, go through the participant information sheet, and confirm their home delivery address for the Garmin watch. The following questions will be asked to confirm participants’ status as a veteran and service history: (1) “Please could you tell me how long you served for?” (2) “And please confirm that you are no longer serving?” (3) “Please could you provide your service number?” and (4) “Please could you tell me a little bit about your time in service? Your role and responsibilities, for example. Just briefly.” Eligible participants who wish to participate in the study will be emailed a copy of the participant information sheet for their own records.

Garmin watches will be posted to participants’ home addresses; participants will be emailed by the research team that the watch is on its way to ensure successful delivery. A video call with a member of the research team will be conducted to support the setup of the device if participants require assistance. Following delivery of the watch, an account will be set up for participants on the SyncRation platform by a member of the research team. Automated emails will be sent from SyncRation to participants’ personal emails throughout the study. A SyncRation automated email (Table 2, SyncRation email 1) will include a link to a document containing participant instructions for setting up the watch and the Garmin Connect app, plus a link to synchronize participants’ Garmin Connect app with the SyncRation portal. This will include consent for the research team to access the participants’ Garmin data via SyncRation. Participants will be requested to begin wearing their watch after they have successfully set up their watch with the Garmin Connect app and consented to the study team accessing their data via the SyncRation platform, to ensure comfort and fit of the device. The research team will be contactable via email to address any technical difficulties throughout the study period.

**Table 2:** Study flowchart—participant journey.

Study phase and activity			Weeks																								
			0		1		2		3		4		5		6		7		8		9		10		11		12
**Recruitment^a^**																											
	Recruitment (Facebook, LinkedIn, veteran groups, or newsletters)																										
	Registration survey: online eligibility screen; contact details: name, email, and phone number; consent to be contacted; participant information sheet; and informed consent.																										
**Onboarding**																											
	Study explanation (verification call)	×																									
	Address confirmed for Garmin delivery (verification call)	×																									
	Veteran status confirmed (verification call)	×																									
	Service history details (verification call)	×																									
	Participant information sheet copy (emailed)	×																									
	Garmin sent to participant’s home address	×																									
	Watch on its way (emailed)	×																									
	Watch and Garmin Connect app setup instructions; Garmin Connect app—SyncRation synchronization link; consent for research team to access data via SyncRation (SyncRation email 1)	×																									
	Baseline measures questionnaire (SyncRation email 2)	×																									
	Optional video call to support setup	×																									
	Day 1—study commencement (emailed)			×																							
**Baseline data**																											
	Sociodemographics	×																									
	International Physical Activity Questionnaire Short Form	×																									
	BMI	×																									
	Dimensions of Anger Reactions-5	×																									
	Insomnia Severity Scale	×																									
	Perceived Stress Scale-10	×																									
**Monthly Mental Health Questionnaire (to be completed every 28 days)**																											
	Alcohol Use Disorders Identification Test for Consumption	×								×								×								×	
	General Health Questionnaire-12	×								×								×								×	
	Generalized Anxiety Disorder Scale-7	×								×								×								×	
	Patient Health Questionnaire-2	×								×								×								×	
	Patient Health Questionnaire-15	×								×								×								×	
	Posttraumatic Stress Disorder checklist for DSM-5^b^	×								×								×								×	
	Pittsburgh Sleep Quality Index	×								×								×								×	
	UCLA^c^ Loneliness Scale-3	×								×								×								×	
	Short Warwick-Edinburgh Mental Well-Being Scale-7	×								×								×								×	
**Fitness tracker**																											
	Sleep: sleep score, sleep hours, and sleep quality			—^d^		—		—		—		—		—		—		—		—		—		—		—	
	HRV^e^ morning and evening			—		—		—		—		—		—		—		—		—		—		—		—	
	Activity: steps, active minutes, and self-reported activity			—		—		—		—		—		—		—		—		—		—		—		—	
	Stress			—		—		—		—		—		—		—		—		—		—		—		—	
	Body battery			—		—		—		—		—		—		—		—		—		—		—		—	
**Questionnaire reminder emails**																											
	MH^f^ Questionnaire link (SyncRation email 3)									×																	
	MH Questionnaire link (SyncRation email 4)																	×									
	MH Questionnaire link + final measures survey (SyncRation email 5)																									×	
**Final measures**																											
	Weight	×																								×	
	Perceived Stress Scale-10	×																								×	
	Insomnia Severity Scale	×																								×	
	Dimensions of Anger Reactions-5	×																								×	
	International Physical Activity Questionnaire Short Form	×																								×	
**Incentive to complete the final questionnaire**																											
	Thank you, plus instructions to unlink Garmin Connect from SyncRation (emailed)																									×	
	£10^g^ Amazon voucher (emailed)																									×	

^a^Recruitment will occur prestudy.

^b^DSM-5: Diagnostic and Statistical Manual of Mental Disorders, Fifth Edition.

^c^UCLA: University of California, Los Angeles.

^d^Continuous data collection from week 1 to week 12.

^e^HRV: heart rate variability.

^f^MH: mental health.

^g^£10=US $13.40.

A link to the baseline measurement questionnaire will be sent to the participants (Table 2, SyncRation email 2). This will include basic demographic information, weight, height, as well as psychometric measures of mental and physical health outcomes. Once the baseline questionnaire is complete, the following day will signify the start date of the study for the participant (Day 1). Participants will complete all monthly questionnaires about their mental and physical health via the online survey platform (Qualtrics). This survey link will be automatically emailed to participants on day 28 (T1), day 56 (T2), and day 84 (T3) of the study via SyncRation. All physiological study data will be collected automatically from the watch, via the Garmin Connect smartphone app. Participants will be emailed 2 reminders to complete each survey: 2 days and 1 week after the survey is first emailed. If the questionnaires remain uncompleted, participants will be called by the research team a further 2 times, 8 days and 10 days after the date the survey link was emailed. Those who still do not complete the survey will be emailed a final time to say they have been withdrawn from the study. Participants who do not complete the questionnaires will not receive the £10 (US $13.40) Amazon gift voucher, but they may opt to keep the Garmin watch. Participants who complete the questionnaires will be gifted their watch after study completion and will receive a £10 (US $13.40) Amazon voucher as a gesture of goodwill for taking part. Participants will be sent a thank-you email for their participation, which will include instructions for unlinking their Garmin Connect app from the SyncRation platform. Participants who do not unlink from SyncRation will be manually unlinked by a member of the research team through the SyncRation platform to ensure cessation of data collection beyond the study end date.

Watches will be delivered to participants’ home addresses and posted in batches. Due to the geographical distribution of participants across the United Kingdom, devices will be received and set up at different time points, resulting in variation in individual study commencement dates (day 1). Study commencement is defined as the point at which the watch is successfully set up and linked to the SyncRation platform and baseline measures are completed. Following successful setup of the device and completion of the baseline questionnaire, participants will be emailed by the research team to mark the study commencement (day 1), and all participants will complete the same study schedule. The study schedule is illustrated in Table 2, which includes all data collection points and questionnaire measures. A selection of health and well-being questionnaires has been selected to enable assessment of participants' mental health and well-being. See Multimedia Appendix 1 for full questionnaire markup.

### Wearable Tracker

Garmin devices (Figure 2) are consumer-grade wearables widely used in health research. Existing validation studies have demonstrated acceptable accuracy for step counting, heart rate, HRV-derived indices, and sleep duration in free-living and laboratory conditions. While these metrics do not replace clinical gold standards, they are considered reasonable proxies for exploratory digital phenotyping studies and have been used in hundreds of published research projects [34]. The device is therefore suitable for feasibility work focusing on patterns of association rather than clinical diagnosis. For this study, the Garmin vívosmart-5 will be used. This device is Conformité Européenne marked and approved for sale and use in the United Kingdom. The device has a battery charge of 7 days and can store data for up to 2 months.

### Sensor Modalities

Sensors included a Garmin Elevate wrist heart rate monitor, Pulse Ox blood oxygen saturation monitor, accelerometer, ambient light sensor (for display brightness adjustment), device-generated values (sleep, stress, maximal oxygen consumption, and zero-crossing), and human-recorded events (activities and body composition; Table 3).

**Table 3:** Garmin vívosmart-5 sensor modalities.

Data type	Variables/description	Sensor
Energy (body battery)	Composite energy score derived from physiological signals	Garmin device (algorithm-generated)
Heart rate	Resting heart rate; heart rate (beats per minute); beat-to-beat interval; heart rate variability	Garmin Elevate wrist heart rate monitor
Respiration	Respiratory rate summaries	Pulse oximeter
Sleep	Sleep score, sleep duration (hours), and sleep quality	Garmin device (algorithm-generated)
SpO_2_^a^	Blood oxygen saturation level	Pulse oximeter
Stress	Physiological stress score derived from heart rate variability	Garmin device (algorithm-generated)
Physical activity	Step count, active minutes, and self-reported activity	Accelerometer; actigraphy; self-reported (human-recorded events)

^a^SpO_2_: saturation peripheral oxygen.

Each smartwatch package includes the following items: Garmin vívosmart-5, USB-C charging cable, and user manual. Participants will be provided with the complete set, consisting of the device and all accompanying contents listed. Participants will be sent instructions on the proper handling and operation of the device. If requested, participants can arrange a video call with a member of the research team to support setup. The manufacturer of the watch, Garmin, will not have access to any study data. At the end of the study, participants can opt to retain the device or send it back to the research team via a prepaid postage box.

### Data Collection

During the study enrollment, participants will be required to download a smartphone app to facilitate data collection (eg, syncing data from the watch). Participants will download the mobile app Garmin Connect via the Apple Store or Google App Store on their personal mobile device. Participants will be required to consent to King’s College London accessing and processing their data via the SyncRation portal. SyncRation is a research-viable product developed by King’s College London to facilitate participant engagement and data synchronization [35]. Participants will be encouraged to synchronize data weekly and at the end of the study via the Garmin Connect app. Participants are able to withdraw at any time via SyncRation, without any need to contact the research team. The app does not collect any personal identifiers such as name, location, or date of birth. The research team will provide the participants with a user manual and guide them through the setup process to explain common features of the app. Once the data are synced, participants will be able to view their data on the Garmin Connect app interface. The research team will be contactable throughout the study to address any technical issues and provide guidance to participants on the use of the vívosmart-5, Garmin Connect app, and SyncRation platform. The research team will not review data in real time but will perform automated quality control checks to ensure data integrity.

### Recruitment

UK veterans will be recruited through convenience sampling methods. Social media posts on LinkedIn, Facebook, and Instagram will advertise the study via open adverts. In addition, veteran groups and peer support networks will be contacted via email and invited to participate in the study (eg, email distribution lists originating from veteran organizations), veteran charities will be contacted, and requests to advertise in relevant charity newsletters will be made. Once participants have successfully completed the eligibility survey and informed consent form, the research assistant will arrange a call with the participant to explain the study in depth, ensure sufficient English language skills, confirm veteran status via service number, and go through the participant information sheet. Participants will be given time to ask questions or raise any concerns. The research assistant will make it clear that participation is entirely voluntary, that participants are not under an obligation to participate, and can withdraw from the study at any time.

### Sample Size

This study is an observational feasibility study. As such, no power calculation has been performed. Research specifically within the UK veteran populations is limited; a sample of 30 was used in a feasibility study using wearable technology to predict PTSD symptoms and cannabis use among UK veterans [36]. Feasibility trials using wearable devices in community and veteran populations commonly recruit between 15 and 110 participants [37-39], and a recent US veteran wearable feasibility study successfully used a sample of 74 [40]. The selected sample size for this study is 50 veterans. A sample of 50 enables the study to estimate key feasibility outcomes, such as recruitment rate, retention, survey completion, and wearable data availability, with acceptable precision for planning a larger study. Although the machine learning (ML) component is exploratory, each participant contributes up to 84 days of continuous data, providing a significant amount of data [40]. In feasibility settings, ML analysis focuses on identifying whether wearable features show consistent signals and whether models can be trained without substantial overfitting, rather than producing validated prediction models.

### Adverse Events

Participation in this study is not expected to result in adverse events. Nevertheless, researchers will monitor changes in participants' mental health over the course of the study to ensure safety. Incoming GHQ-12 scores from participants' monthly surveys will be monitored on a weekly basis by the research team. Weekly monitoring will be conducted to account for participants’ different start dates and, therefore, different monthly questionnaire completion dates between participants, to ensure that safeguarding response procedures are initiated within a week of data collection. Any participants reporting an increase in GHQ-12 score of ≥15 points between 2 consecutive surveys will be contacted immediately by a member of the research team. During this contact, participants will be provided with the signposting booklet of veterans’ mental health services. If concerns about the participant’s well-being arise during this correspondence, the case will be escalated for further assessment. This will involve a call with a designated member of the research team. Further action required following confirmation of risk will result in a referral to the clinical lead (DM) to perform a clinical interview and risk assessment, and to determine whether and what additional support is required and whether the participant should be withdrawn from the study. All contact, assessments, and actions undertaken in response to potential adverse events will be documented, and any data collected before withdrawal will be used for analysis.

### Statistical Analysis

#### Overview

Analyses will be conducted after data collection. As this is a feasibility study, the primary analytic aims are to describe data availability, engagement, and retention, and to explore preliminary associations between wearable-derived features and mental health outcomes. All analyses will be exploratory and will inform the design of a future larger study.

#### Descriptive and Feasibility Analyses

Baseline characteristics will be summarized using standard descriptive statistics. Questionnaire outcomes will be described at T0, T1, T2, and T3. Feasibility outcomes (recruitment rate, retention, questionnaire completion, proportion of valid wear days and weeks, and completeness of each wearable stream) will be reported using counts, percentages, medians, and IQRs as appropriate. Participants will be grouped into quartiles of data completeness to assess engagement patterns as undertaken in prior work [40,41].

#### Wearable Feature Construction

Wearable data (steps, active minutes, sleep metrics, heart rate, HRV, stress, and body battery) will be summarized at the day and week level. Features will include mean values, variability measures, and simple temporal trends. Final feature inclusion for ML analysis will depend on data completeness. All sensor-derived features will be treated as exploratory physiological indices and not as validated clinical biomarkers.

#### Missing Data

For wearable data, a valid day will be defined as having at least 6 hours of data for each individual stream. Weekly summaries will require at least 4 valid days. Missing days and weeks will not be imputed in the primary analysis. Patterns of missingness will be summarized and reported.

#### Exploratory Modelling

The primary exploratory outcome is the change in GHQ-12 score from T0 to T3. Secondary outcomes include change in other questionnaire scores. Initial associations between wearable features and mental health outcomes will be examined using simple regression models to estimate effect sizes.

#### ML Analysis

Candidate models will include a range of classifiers such as decision trees, random forests, support vector machines, and Bayesian classifiers. To reduce information leakage arising from repeated measures, models will be evaluated using participant-level grouped k-fold cross-validation, such that all observations from a given participant are assigned to either the training or test set within each fold, but not both. Hyperparameter tuning will be conducted within the training data only. Performance metrics will include area under the curve, sensitivity, and specificity for binary outcomes. ML analysis will be judged informative if (1) models can be fitted without substantial overfitting, (2) performance exceeds simple baseline models, and (3) a small number of wearable features consistently appear influential across models. Feature importance will be used to identify suitable data streams for a future larger study.

## Results

Enrollment for the pilot phase was completed in December 2024, with all pilot study participants completing the study (n=5). Data collection for the main study commenced in February 2025 and concluded in October 2025. As of March 2026, 52 participants have been recruited, of whom 48 have completed the study procedure, and 4 have withdrawn from the study. Recruitment occurred between February 2025 and August 2025. Data analysis is scheduled to begin in January 2026, with results expected to be submitted for publication in May 2026.

## Discussion

### Overview

This study protocol outlines the design for the V-MIND (Veteran Monitoring Initiative for Noninvasive Physiology and Depression) observational digital feasibility study. A longitudinal 3-month (84-day) study designed to observe changes in participant mental health and physiological metrics (eg, physical activity, sleep, and stress) collected continuously via Garmin vívosmart-5 wearable devices. The participant sample includes 48 UK veterans who met caseness for CMDs, including symptoms of anxiety and depression measured by the GHQ-12.

Findings from this study could provide essential information on the feasibility of using wearables to collect continuous physiological data alongside self-reported mental health measures in a UK veteran population. The study will evaluate whether participants can be recruited at a rate sufficient to support a future larger participant group. We hypothesize that this study can be implemented without major protocol deviations, and successful implementation will be considered as a measure of operational feasibility of the V-MIND study. It is expected that key outcomes will demonstrate sufficient variability rates to inform a sample size calculation for a potentially larger-scale future study, and that wearable-derived metrics associated with mental health outcomes will be observed.

The value of this work could be realized in the development of novel interventions used to manage veteran mental health. Digital interventions have been used to encourage self-disclosure of mental health issues to a professional among civilians [42], and there is potential scope to combine similar initiatives with wearable technology for veterans. The feasibility and acceptability of technology-driven methods for use with mental health treatment–seeking veteran groups have been demonstrated in previous work [19], which identified digital interventions as a promising avenue for veteran health initiatives. Other studies have also demonstrated this. For example, a randomized controlled trial found that using Garmin wearables (fēnix 5, fēnix 6, and vívoactive 4 series) and mobile health (mHealth) apps alongside cognitive behavioral therapy (CBT) improved mental health disorder symptoms among active-duty US service members [43]. In the case of CBT, adherence is largely associated with therapeutic success [44]. In this study, 50% of the control group (n=5) dropped out of CBT early; those who used the mHealth app and wearable device completed therapy and showed reductions in mental health symptoms, including depression, anxiety, stress, and anger. Results suggest wearables may improve treatment compliance among military personnel, and other studies have identified similar increased adherence to therapy among veteran groups when they used an mHealth adjunct [45]. Due to high rates of dropout during therapy, 42% on average for veterans receiving clinical care, increasing to 68% for veterans undergoing treatment for PTSD [13,46], the use of wearable technology as a study tool, as a potential treatment adjuvant, or for symptom monitoring purposes may be particularly relevant to this population. Commercially available, discreet, and inherently unobtrusive wearable devices offer avenues for veterans to engage with their mental health outside of formal clinical routes. Devices such as Fitbit have, for example, been identified as an acceptable adjunct in behavioral activation therapy for depression among civilian samples [47]. The full potential of wearables in mental health care settings is yet to be established, and research is ongoing among veteran and other general population groups [48].

Garmin Vivofit-4 devices have been identified as feasible for use as a self-monitoring tool in a study exploring problem anger in trauma-exposed civilians [49]. Reductions in anger symptoms were observed in this group, alongside PTSD symptom improvements following use of a smartphone app which prompted participants to reflect on their anger in conjunction with the wearable device, which alerted participants when it measured high levels of physiological stress. Although limited by the lack of a control group and a majority female participants sample, this work made important contributions to the evidence base exploring the potential for wearables to be leveraged in novel digital health interventions.

Physical activity and functioning factors such as sleep and stress have been collected using wearable devices alongside mobile sensing data to explore mental health and well-being outcomes among other occupational groups. One such study combined Fitbit data, including measures such as heart rate, with short self-reported surveys delivered to participants’ smartphones, to predict indicators of stress resilience in US medical interns [50]. Evidencing the use of technology-driven health monitoring for groups under unique occupational stress. Other studies have also used wearable devices and mobile sensing data to predict clinically significant changes in mental health, including depression [51], anxiety [52], and bipolar disorder [53] among general population samples. WHOOP devices, for example, have been used to observe the relationship between mental health and wearable metrics such as HRV and found that participants with better sleep, higher HRV, lower resting heart rate, and higher physical activity levels reported lower depression, anxiety, and stress scores. Interpersonal analysis showed that physiological indices collected via WHOOP devices were associated with self-reported mental health outcomes [54]. However, this work has yet to be replicated in veteran populations.

### Strengths and Limitations

Study strengths include the use of a smartphone app, which enables remote data collection from veteran participants across the United Kingdom. Participants will be sent the watch as part of the study; they are required to have a smartphone and access to a USB-C charging port, which potentially restricts the pool of participants. Wrist-worn devices may be particularly beneficial for use with veteran population because they are discrete, meaning they have the potential to overcome some of the concerns veterans may have about seeking help for mental health difficulties such as stigma, which are consistently identified as a major barrier to help-seeking among veterans [5]. Although wearables can provide research-quality data, accuracy is a concern. When compared with polysomnography, for example, the gold standard for assessing sleep, wearables fall short. A systematic review comparing the performance of wearables (Fitbit Charge 4, Garmin vívosmart-4, and WHOOP) in measuring participant sleep found that WHOOP performed better as a measure of total sleep time compared to the other models, although Garmin vívosmart-4 and Fitbit Charge 4 showed moderate accuracy in measuring sleep stages and total sleep time [55]. Polysomnography is impractical for everyday use; it is expensive, requires specialist equipment and expertise and is not feasible for population level interventions. In contrast, wearables such as the vivosmart-5 are commercially available and better suited to day-to-day use. Therefore, a limitation is that Garmin metrics are derived from proprietary algorithms and have variable validation against clinical gold-standard measures. As such, findings will be interpreted as exploratory signals, and not as diagnostic physiological outputs. The decision to recruit participants solely through social media platforms and veteran community groups may introduce sample bias and limit generalizability to the broader population, as this potentially excludes those with limited digital access or low comfort with technology. This may need to be considered when interpreting the study findings and planning a larger, more representative future trial.

### Conclusion

This study aims to contribute to the developing evidence-based exploring mHealth approaches and the use of wearable technology in veteran mental health research. Findings will provide valuable data on the feasibility of using wearables to collect continuous physiological data alongside self-reported mental health measures in a UK veteran population. The study will examine associations between CMDs, including symptoms of anxiety and depression, and physical activity indices in UK veterans. While such associations have been explored within civilian populations, they remain largely under investigated among specific occupational groups. Although modest in size (n=50), this study will allow for the observation of key associations within the analysis, and findings may inform a larger future trial. This work could support the development of interventions that integrate wearable-derived data to enhance the prediction and management of CMDs in UK veteran populations.

## Appendix

Multimedia Appendix 1 Measures.

## Abbreviations

CBT: cognitive behavioral therapy
CMD: common mental disorder
GDPR: General Data Protection Regulation
GHQ-12: 12-item General Health Questionnaire
HRV: heart rate variability
mHealth: mobile health
ML: machine learning
PTSD: posttraumatic stress disorder
V-MIND: Veteran Monitoring Initiative for Noninvasive Physiology and Depression

## References

[1] Rhead R, MacManus D, Jones M, Greenberg N, Fear NT, Goodwin L (2022). Mental health disorders and alcohol misuse among UK military veterans and the general population: a comparison study. Psychol Med.

[2] (2018). The strategy for our veterans. HM Government.

[3] (2021). Characteristics of UK Armed Forces veterans, England and Wales. Office for National Statistics.

[4] Scotland’s Census 2022 - UK Armed Forces veterans. Scotland's Census.

[5] Census 2021 topic report on UK Armed Forces veterans. Northern Ireland Statistics and Research Agency.

[6] El-Gabalawy R, Blaney C, Tsai J, Sumner JA, Pietrzak RH (2018). Physical health conditions associated with full and subthreshold PTSD in U.S. military veterans: results from the national health and resilience in veterans study. J Affect Disord.

[7] Risks to mental health. World Health Organisation.

[8] Liu D, McIntyre RS, Li R, Yang M, Xue Y, Cao B (2021). Genetic association between major depressive disorder and type 2 diabetes mellitus: shared pathways and protein networks. Prog Neuropsychopharmacol Biol Psychiatry.

[9] Williamson C, Palmer L, Leightley D, Pernet D, Chandran D, Leal R, Murphy D, Fear NT, Stevelink SAM (2023). Military veterans and civilians' mental health diagnoses: an analysis of secondary mental health services. Soc Psychiatry Psychiatr Epidemiol.

[10] McDaid D, Park AL The economic case for investing in the prevention of mental health conditions in the UK (summary). Mental Health Foundation.

[11] Mark KM, Leightley D, Pernet D, Murphy D, Stevelink SAM, Fear NT (2019). Identifying veterans using electronic health records in the United Kingdom: a feasibility study. Healthcare (Basel).

[12] Sharp ML, Franchini S, Jones M (2024). Health and wellbeing study of serving and ex-serving UK Armed Forces personnel: phase 4 (Office for Veterans' Affairs final report). King's College London.

[13] Amsalem D, Lopez-Yianilos A, Lowell A, Pickover AM, Arnon S, Zhu X, Suarez-Jimenez B, Ryba M, Bergman M, Such S, Zalman H, Sanchez-Lacay A, Lazarov A, Markowitz JC, Neria Y (2022). Treatment dropout among veterans and their families: quantitative and qualitative findings. Psychol Trauma.

[14] Lee S, Kim H, Park MJ, Jeon HJ (2021). Current advances in wearable devices and their sensors in patients with depression. Front Psychiatry.

[15] Moshe I, Terhorst Y, Philippi P, Domhardt M, Cuijpers P, Cristea I, Pulkki-Råback L, Baumeister H, Sander LB (2021). Digital interventions for the treatment of depression: a meta-analytic review. Psychol Bull.

[16] Li K, Cardoso C, Moctezuma-Ramirez A, Elgalad A, Perin E (2023). Heart rate variability measurement through a smart wearable device: another breakthrough for personal health monitoring?. Int J Environ Res Public Health.

[17] Zhang Y, Folarin AA, Sun S, Cummins N, Ranjan Y, Rashid Z, Stewart C, Conde P, Sankesara H, Laiou P, Matcham F, White KM, Oetzmann C, Lamers F, Siddi S, Simblett S, Vairavan S, Myin-Germeys I, Mohr DC, Wykes T, Haro JM, Annas P, Penninx BW, Narayan VA, Hotopf M, Dobson RJ, RADAR-CNS consortium (2024). Longitudinal assessment of seasonal impacts and depression associations on circadian rhythm using multimodal wearable sensing: retrospective analysis. J Med Internet Res.

[18] Huhn S, Axt M, Gunga H, Maggioni MA, Munga S, Obor D, Sié Ali, Boudo V, Bunker A, Sauerborn R, Bärnighausen Till, Barteit S (2022). The impact of wearable technologies in health research: scoping review. JMIR Mhealth Uhealth.

[19] Leightley D, Williamson C, Rona RJ, Carr E, Shearer J, Davis JP, Simms A, Fear NT, Goodwin L, Murphy D (2022). Evaluating the efficacy of the drinks: Ration mobile app to reduce alcohol consumption in a help-seeking military veteran population: randomized controlled trial. JMIR Mhealth Uhealth.

[20] Burdett H, Woodhead C, Iversen AC, Wessely S, Dandeker C (2013). “Are you a veteran?” understanding of the term “veteran” among UK ex-service personnel: a research note. Armed Forces Soc.

[21] Goldberg D (1988). A User's Guide to the General Health Questionnaire.

[22] Bush K, Kivlahan DR, McDonell MB, Fihn SD, Bradley KA (1998). The AUDIT alcohol consumption questions (AUDIT-C): an effective brief screening test for problem drinking. Ambulatory Care Quality Improvement Project (ACQUIP). Alcohol use disorders identification test. Arch Intern Med.

[23] Spitzer RL, Kroenke K, Williams JBW, Löwe B (2006). A brief measure for assessing generalized anxiety disorder: the GAD-7. Arch Intern Med.

[24] Kroenke K, Spitzer RL, Williams JBW (2003). The Patient Health Questionnaire-2: validity of a two-item depression screener. Med Care.

[25] Kroenke K, Spitzer RL, Williams JBW (2002). The PHQ-15: validity of a new measure for evaluating the severity of somatic symptoms. Psychosom Med.

[26] Zuromski KL, Ustun B, Hwang I, Keane TM, Marx BP, Stein MB, Ursano RJ, Kessler RC (2019). Developing an optimal short-form of the PTSD Checklist for DSM-5 (PCL-5). Depress Anxiety.

[27] Buysse DJ, Reynolds CF, Monk TH, Berman SR, Kupfer DJ (1989). The Pittsburgh Sleep Quality Index: a new instrument for psychiatric practice and research. Psychiatry Res.

[28] Russell DW (1996). UCLA Loneliness Scale (version 3): reliability, validity, and factor structure. J Pers Assess.

[29] Shah N, Cader M, Andrews B, McCabe R, Stewart-Brown SL (2021). Short Warwick-Edinburgh Mental Well-Being Scale (SWEMWBS): performance in a clinical sample in relation to PHQ-9 and GAD-7. Health Qual Life Outcomes.

[30] Forbes D, Alkemade N, Hopcraft D, Hawthorne G, O'Halloran P, Elhai JD, McHugh T, Bates G, Novaco RW, Bryant R, Lewis V (2014). Evaluation of the Dimensions of Anger Reactions-5 (DAR-5) scale in combat veterans with posttraumatic stress disorder. J Anxiety Disord.

[31] Bastien CH, Vallières A, Morin CM (2001). Validation of the Insomnia Severity Index as an outcome measure for insomnia research. Sleep Med.

[32] Craig CL, Marshall AL, Sjöström M, Bauman AE, Booth ML, Ainsworth BE, Pratt M, Ekelund U, Yngve A, Sallis JF, Oja P (2003). International Physical Activity Questionnaire: 12-country reliability and validity. Med Sci Sports Exerc.

[33] Cohen S, Kamarck T, Mermelstein R (1983). A global measure of perceived stress. J Health Soc Behav.

[34] 2021-2025 current years | Garmin health third-party studies. Garmin.

[35] Leightley D (2024). Navigating the software development landscape: introducing the term research viable product (RVP). OSF.

[36] Williamson G, Trompeter N, Murphy D, Saba S, Pedersen ER, Davis JP, Leightley D (2024). Using passive and active data to predict post-traumatic stress disorder symptoms and cannabis use in recently discharged UK Veterans: a protocol for the MAVERICK feasibility study. Mental Health Sci.

[37] Kim RH, Patel MS (2018). Barriers and opportunities for using wearable devices to increase physical activity among veterans: pilot study. JMIR Form Res.

[38] Nakagome K, Makinodan M, Uratani M, Kato M, Ozaki N, Miyata S, Iwamoto K, Hashimoto N, Toyomaki A, Mishima K, Ogasawara M, Takeshima M, Minato K, Fukami T, Oba M, Takeda K, Oi H (2023). Feasibility of a wrist-worn wearable device for estimating mental health status in patients with mental illness. Front Psychiatry.

[39] Saleem JJ, Wilck NR, Murphy JJ, Herout J (2022). Veteran and staff experience from a pilot program of health care system-distributed wearable devices and data sharing. Appl Clin Inform.

[40] Leightley D, Dilkina B, Pedersen ER, Dworkin E, Saba S, Howe E, Thota P, Nuthi S, Sedano A, Davis JP (2025). A remote measurement study of PTSD and cannabis use among veterans: recruitment, retention, and data availability. PLoS One.

[41] Matcham F, Leightley D, Siddi S, Lamers F, White KM, Annas P, de Girolamo G, Difrancesco S, Haro JM, Horsfall M, Ivan A, Lavelle G, Li Q, Lombardini F, Mohr DC, Narayan VA, Oetzmann C, Penninx BWJH, Bruce S, Nica R, Simblett SK, Wykes T, Brasen JC, Myin-Germeys I, Rintala A, Conde P, Dobson RJB, Folarin AA, Stewart C, Ranjan Y, Rashid Z, Cummins N, Manyakov NV, Vairavan S, Hotopf M, RADAR-CNS consortium (2022). Remote assessment of disease and relapse in major depressive disorder (RADAR-MDD): recruitment, retention, and data availability in a longitudinal remote measurement study. BMC Psychiatry.

[42] Lee Y, Yamashita N, Huang Y (2020). Designing a chatbot as a mediator for promoting deep self-disclosure to a real mental health professional. Proc ACM Hum Comput Interact.

[43] Winslow BD, Kwasinski R, Hullfish J, Ruble M, Lynch A, Rogers T, Nofziger D, Brim W, Woodworth C (2022). Automated stress detection using mobile application and wearable sensors improves symptoms of mental health disorders in military personnel. Front Digit Health.

[44] Decker SE, Kiluk BD, Frankforter T, Babuscio T, Nich C, Carroll KM (2016). Just showing up is not enough: homework adherence and outcome in cognitive-behavioral therapy for cocaine dependence. J Consult Clin Psychol.

[45] Winslow BD, Chadderdon GL, Dechmerowski SJ, Jones DL, Kalkstein S, Greene JL, Gehrman P (2016). Development and clinical evaluation of an mHealth application for stress management. Front Psychiatry.

[46] Goetter EM, Bui E, Ojserkis RA, Zakarian RJ, Brendel RW, Simon NM (2015). A systematic review of dropout from psychotherapy for posttraumatic stress disorder among Iraq and Afghanistan combat veterans. J Trauma Stress.

[47] Chum J, Kim MS, Zielinski L, Bhatt M, Chung D, Yeung S, Litke K, McCabe K, Whattam J, Garrick L, O'Neill L, Goyert S, Merrifield C, Patel Y, Samaan Z (2017). Acceptability of the Fitbit in behavioural activation therapy for depression: a qualitative study. Evid Based Ment Health.

[48] Borghare PT, Methwani DA, Pathade AG (2024). A comprehensive review on harnessing wearable technology for enhanced depression treatment. Cureus.

[49] Metcalf O, Pham L, Lamb KE, Zaloumis S, O'Donnell ML, Qian T, Varker T, Cowlishaw S, Forbes D (2025). A mixed-methods investigation of a digital mental health tool to manage posttrauma anger. J Trauma Stress.

[50] Adler DA, Tseng VW, Qi G, Scarpa J, Sen S, Choudhury T (2021). Identifying mobile sensing indicators of stress-resilience. Proc ACM Interact Mob Wearable Ubiquitous Technol.

[51] Wang R, Wang W, Dasilva A, Huckins JF, Kelley WM, Heatherton TF, Campbell AT (2018). Tracking depression dynamics in college students using mobile phone and wearable sensing. Proc ACM Interact Mob Wearable Ubiquitous Technol.

[52] Jacobson NC, Bhattacharya S (2022). Digital biomarkers of anxiety disorder symptom changes: personalized deep learning models using smartphone sensors accurately predict anxiety symptoms from ecological momentary assessments. Behav Res Ther.

[53] Lipschitz JM, Lin S, Saghafian S, Pike CK, Burdick KE (2025). Digital phenotyping in bipolar disorder: using longitudinal Fitbit data and personalized machine learning to predict mood symptomatology. Acta Psychiatr Scand.

[54] Presby D, Jasinski S, Capodilupo E, Holmes KE, von Hippel W, Grosicki GJ, Lee V (2025). Inter- and intrapersonal associations between physiology and mental health: a longitudinal study using wearables and mental health surveys. J Med Internet Res.

[55] Schyvens AM, Van Oost NC, Aerts JM, Masci F, Peters B, Neven A, Dirix H, Wets G, Ross V, Verbraecken J (2024). Accuracy of Fitbit charge 4, Garmin Vivosmart 4, and WHOOP versus polysomnography: systematic review. JMIR Mhealth Uhealth.

