# Supplementary material for: Veteran Monitoring Initiative for Noninvasive Physiology and Depression (V-MIND) Exploring Physical Activity and Mental Health in UK Veterans: Protocol for an Observational Digital Phenotyping Study
Source: JMIR Res Protoc. 2026 Apr 29;15:e73060. doi: 10.2196/73060 (PMC13173092; doi:10.2196/73060)
Supplement: Multimedia Appendix 1 [file resprot_v15i1e73060_app1.docx]

| Question | Scoring System | | | | |
| --- | --- | --- | --- | --- | --- |
|  | 0 | 1 | 2 | 3 | 4 |
| 1) How often do you have a drink containing alcohol? | Never | Monthly or less | 2 to 4 times per month | 2 to 3 times per week | 4 or more times per week |
| 2) How many units of alcohol do you drink on a typical day when you are drinking? | 0-2 | 3-4 | 5-6 | 7-9 | 10 or more |
| 3) How often have you had 6 or more units if female, or 8 or more if male, on a single occasion in the last year? | Never | Less than monthly | Monthly | Weekly | Daily or almost daily |

**
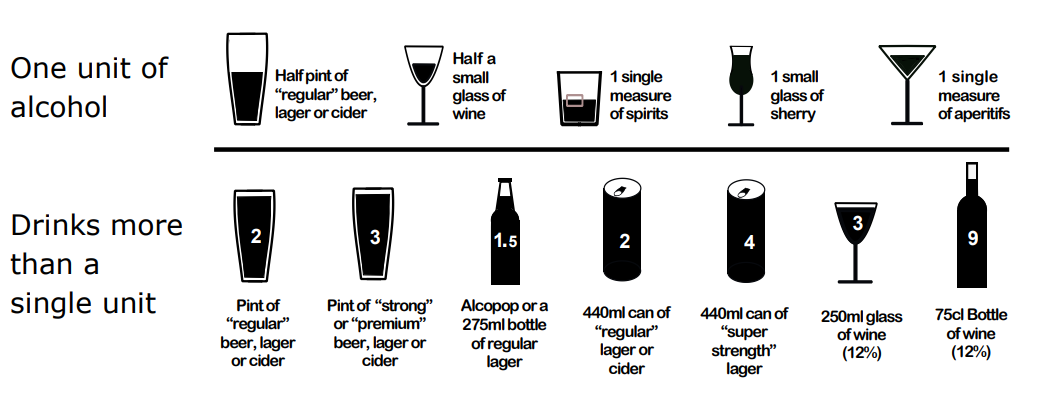
Alcohol use Disorders identification Test for Consumption (AUDIT-C)**Alcohol unit reference: **One unit of alcohol


Drinks more than one unit of alcohol**

**Body Mass Index (BMI)**

| 1) Please enter your weight in kilograms [Free text] |
| --- |
| 2) Please enter your height in metres [Free text] |

**DAR-5 (Dimensions of Anger Reactions-5)**Thinking over the past 4 weeks, select the option that best describes the amount of time you felt that way.

| Questions | Score | | | | |
| --- | --- | --- | --- | --- | --- |
|  | 1 | 2 | 3 | 4 | 5 |
| 1) I found myself getting angry at people or situations | None or almost none of the time | A little of the time | Some of the time | Most of the time | All or almost all of the time |
| 2) When I got angry, I got really mad | None or almost none of the time | A little of the time | Some of the time | Most of the time | All or almost all of the time |
| 3) When I got angry, I stayed angry | None or almost none of the time | A little of the time | Some of the time | Most of the time | All or almost all of the time |
| 4) When I got angry at someone I wanted to hit them | None or almost none of the time | A little of the time | Some of the time | Most of the time | All or almost all of the time |
| 5) My anger prevented me from getting along with people as well as I’d have liked to | None or almost none of the time | A little of the time | Some of the time | Most of the time | All or almost all of the time |

**General health Questionnaire (GHQ-12)**

Within **the past month,** have you…

| Question | Score | | | |
| --- | --- | --- | --- | --- |
|  | 0 | 1 | 2 | 3 |
| 1) Been able to concentrate on what you’re doing? | Better than usual | Same as usual | Less than usual | Much less than usual |
| 2) Lost much sleep due to some worry? | Not at all | No more than usual | Rather more than usual | Much more than usual |
| 3) Felt you were playing a useful part in things? | More so than usual | Same as usual | Less useful than usual | Much less useful than usual |
| 4) Felt capable of making decisions about things? | More so than usual | Same as usual | Less so than usual | Much less capable than usual |
| 5) Felt constantly under strain? | Not at all | No more than usual | Rather more than usual | Much more than usual |
| 6) Felt you couldn’t overcome your difficulties? | Not at all | No more than usual | Rather more than usual | Much more than usual |
| 7) Been able to enjoy your normal day-to-day activities? | More so than usual | Same as usual | Less than usual | Much less than usual |
| 8) Been able to face up to your problems? | More so than usual | Same as usual | Less able than usual | Much less able than usual |
| 9) Been feeling unhappy and depressed? | Not at all | No more than usual | Rather more than usual | Much more than usual |
| 10) Been losing confidence in yourself? | Not at all | No more than usual | Rather more than usual | Much more than usual |
| 11) Been thinking of yourself as a worthless person? | Not at all | No more than usual | Rather more than usual | Much more than usual |
| 12) Been feeling reasonably happy, all things considered | More so than usual | Same as usual | Less than usual | Much less than usual |

**Generalised Anxiety Disorder (GAD-7)**

Over the last two weeks, how often have you been bothered by the following problems?

| Question | Score | | | |
| --- | --- | --- | --- | --- |
|  | 0 | 1 | 2 | 3 |
| 1) Feeling nervous, anxious or on edge | Not at all | Several days | More than half the days | Nearly every day |
| 2) Not being able to stop or control worrying | Not at all | Several days | More than half the days | Nearly every day |
| 3) Worrying too much about different things | Not at all | Several days | More than half the days | Nearly every day |
| 4) Trouble relaxing | Not at all | Several days | More than half the days | Nearly every day |
| 5) Being so restless that it is hard to sit still | Not at all | Several days | More than half the days | Nearly every day |
| 6) Becoming easily annoyed or irritable | Not at all | Several days | More than half the days | Nearly every day |
| 7) Feeling afraid, as if something awful might happen | Not at all | Several days | More than half the days | Nearly every day |

If you checked off any problems, how difficult have these made it for you to do your work, take care of things at home, or get along with other people?

| Not difficult at all | Somewhat difficult | Very Difficult | Extremely Difficult |
| --- | --- | --- | --- |

**Insomnia Severity Index (ISI)**

Please rate the current **(i.e., last two weeks)** severity of your insomnia problems

| Question | Score | | | | |
| --- | --- | --- | --- | --- | --- |
|  | 0 | 1 | 2 | 3 | 4 |
| 1) Difficulty falling asleep | None | Mild | Moderate | Severe | Very severe |
| 2) Difficulty staying asleep | None | Mild | Moderate | Severe | Very severe |
| 3) Problems waking up too early | None | Mild | Moderate | Severe | Very severe |
| 4) How satisfied/dissatisfied are you with your **current** sleep pattern? | Very satisfied | Satisfied | Moderately satisfied | Dissatisfied | Very dissatisfied |
| 5) How noticeable to others do you think your sleep problem is in terms of impairing the quality of your life? | Not at all noticeable | A little | Somewhat | Much | Very much noticeable |
| 6) How worried/distressed are you about your current sleep problem? | Not at all worried | A little | Somewhat | Much | Very much worried |
| 7) To what extent do you consider your sleep problem to interfere with your daily functioning (e.g., daytime fatigue, mood, ability to functioning at work/daily chores, concentration, memory, mood, etc.) currently? | Not at all interfering | A little | Somewhat | Much | Very much interfering |

**International Physical Activity Questionnaire - Short Form (IPAQ-SF)**These questions will ask you about the time you spent being physically active in the last 7 days. Please answer each question even if you do not consider yourself to be an active person. Please think about the activities you do at work, as part of your house and yard work, to get from place to place, and in your spare time for recreation, exercise or sport.

Think about all the vigorous activities that you did in the last 7 days. Vigorous physical activities refer to activities that take hard physical effort and make you breathe much harder than normal. Think only about those physical activities that you did for at least 10 minutes at a time.

| 1) During the last 7 days, on how many days did you do vigorous physical activities like heavy lifting, digging, aerobics, or fast bicycling? | Days per week _______ | No Vigorous physical activity ^1^ |
| --- | --- | --- |
| 2) How much time did you usually spend doing vigorous physical activities on one of those days? | Hours per day _____ | |
|  | Minutes per day _____ | |
|  | Don’t know/not sure | |

^1^ If selected, skip to question 3

Think about all the moderate activities that you did in the last 7 days. Moderate activities refer to activities that take moderate physical effort and make you breathe somewhat harder than normal. Think only about those physical activities that you did for at least 10 minutes at a time

| 3) During the last 7 days, on how many days did you do moderate physical activities like carrying light loads, bicycling at a regular pace, or doubles tennis? Do not include walking. | Days per week _______ | No Vigorous physical activity ^2^ |
| --- | --- | --- |
| 4) How much time did you usually spend doing moderate physical activities on one of those days? | Hours per day _____ | |
|  | Minutes per day _____ | |
|  | Don’t know/not sure | |

^2^ If selected, skip to question 5

Think about the time you spent walking in the last 7 days. This includes at work and at home, walking to travel from place to place, and any other walking that you have done solely for recreation, sport, exercise, or leisure.

| 5) During the last 7 days, on how many days did you walk for at least 10 minutes at a time? | Days per week _______ | No walking ^3^ |
| --- | --- | --- |
| 6) How much time did you usually spend walking on one of those days? | Hours per day _____ | |
|  | Minutes per day _____ | |
|  | Don’t know/not sure | |

^3^ If selected, skip to question 7

The last question is about the time you spent sitting on weekdays during the last 7 days. Include time spent at work, at home, while doing course work and during leisure time. This may include time spent sitting at a desk, visiting friends, reading, or sitting or lying down to watch television.

| 7) How much time did you spend sitting on a week day? | Hours per day _____ |
| --- | --- |
|  | Minutes per day _____ |
|  | Don’t know/not sure |

**Patient Health Questionnaire (PHQ-2)**

Over the past two weeks, how often have you been bothered by any of the following problems?

| Question | Score | | | |
| --- | --- | --- | --- | --- |
|  | 0 | 1 | 2 | 3 |
| Little interest or pleasure in doing things | Not at all | Several Days | More than half the days | Nearly every day |
| Feeling down, depressed, or hopeless | Not at all | Several Days | More than half the days | Nearly every day |

**Patient Health Questionnaire-15 (PHQ-15)**

During the **past 4 weeks**, how much have you been bothered by the following problems?

| 1) Stomach pain | Not bothered at all | Bothered a little bit | Bothered a lot |
| --- | --- | --- | --- |
| 2) Back pain | Not bothered at all | Bothered a little bit | Bothered a lot |
| 3) Pain in your arms, legs, or joints (knees, hips, etc.) | Not bothered at all | Bothered a little bit | Bothered a lot |
| 4) Menstrual cramps or other problems with your periods Please leave blank if this does not apply. | Not bothered at all | Bothered a little bit | Bothered a lot |
| 5) Headaches | Not bothered at all | Bothered a little bit | Bothered a lot |
| 6) Chest pains | Not bothered at all | Bothered a little bit | Bothered a lot |
| 7) Dizziness | Not bothered at all | Bothered a little bit | Bothered a lot |
| 8) Fainting spells | Not bothered at all | Bothered a little bit | Bothered a lot |
| 9) Feeling your heart pound or race | Not bothered at all | Bothered a little bit | Bothered a lot |
| 10) Shortness of breath | Not bothered at all | Bothered a little bit | Bothered a lot |
| 11) Pain or problems during sexual intercourse | Not bothered at all | Bothered a little bit | Bothered a lot |
| 12) Constipation, loose bowels, or diarrhea | Not bothered at all | Bothered a little bit | Bothered a lot |
| 13) Nausea, gas, or indigestion | Not bothered at all | Bothered a little bit | Bothered a lot |
| 14) Feeling tired or having low energy | Not at all | Several days | More than half the days |
| 15) Trouble sleeping | Not at all | Several days | More than half the days |

**Perceived Stress Scale (PSS-10)**

| Question | Score | | | | |
| --- | --- | --- | --- | --- | --- |
|  | 0 | 1 | 2 | 3 | 4 |
| 1) In the last month, how often have you been upset because of something that happened unexpectedly? | Never | Almost never | Sometimes | Fairly often | Very often |
| 2) In the last month, how often have you felt that you were unable to control the important things in your life? | Never | Almost never | Sometimes | Fairly often | Very often |
| 3) In the last month, how often have you felt nervous and stressed? | Never | Almost never | Sometimes | Fairly often | Very often |
| 4) In the last month, how often have you felt confident about your ability to handle your personal problems? | Never | Almost never | Sometimes | Fairly often | Very often |
| 5) In the last month, how often have you felt that things were going your way? | Never | Almost never | Sometimes | Fairly often | Very often |
| 6) In the last month, how often have you found that you could not cope with all the things that you had to do? | Never | Almost never | Sometimes | Fairly often | Very often |
| 7) In the last month, how often have you been able to control irritations in your life? | Never | Almost never | Sometimes | Fairly often | Very often |
| 8) In the last month, how often have you felt that you were on top of things? | Never | Almost never | Sometimes | Fairly often | Very often |
| 9) In the last month, how often have you been angered because of things that happened that were outside of your control? | Never | Almost never | Sometimes | Fairly often | Very often |
| 10) In the last month, how often have you felt difficulties were piling up so high that you could not overcome them? | Never | Almost never | Sometimes | Fairly often | Very often |

**PTSD Checklist for DSM-IV (PCL-5)**

Below is a list of problems that some people have in response to a very stressful experience. While keeping your most stressful experience in mind, please read each problem carefully and then indicate how much you have been bothered by that problem in the past month.

In the **past month,** how much were you bothered by:

| Question | Score | | | | |
| --- | --- | --- | --- | --- | --- |
|  | 0 | 1 | 2 | 3 | 4 |
| 1) Repeated, disturbing, and unwanted memories of the stressful experience? | Not at all | A little bit | Moderately | Quite a bit | Extremely |
| 2) Repeated, disturbing dreams of the stressful experience? | Not at all | A little bit | Moderately | Quite a bit | Extremely |
| 3) Suddenly feeling or acting as if the stressful experience were actually happening again (as if you were actually back there reliving it)? | Not at all | A little bit | Moderately | Quite a bit | Extremely |
| 4) Feeling very upset when something reminded you of the stressful experience? | Not at all | A little bit | Moderately | Quite a bit | Extremely |
| 5) Having strong physical reactions when something reminded you of the stressful experience (for example, heart pounding, trouble breathing, sweating) | Not at all | A little bit | Moderately | Quite a bit | Extremely |
| 6) Avoiding memories, thoughts, or feelings related to the stressful experience? | Not at all | A little bit | Moderately | Quite a bit | Extremely |
| 7) Avoiding external reminders of the experience (for example, people, places, conversations, objects, activities, or situations)? | Not at all | A little bit | Moderately | Quite a bit | Extremely |
| 8) Trouble remembering important parts of the stressful experience? | Not at all | A little bit | Moderately | Quite a bit | Extremely |
| 9) Having strong negative beliefs about yourself, other people, or the world (for example, having thoughts such as: I am bad, there is something seriously wrong with me, no one can be trusted, the world is completely dangerous)? | Not at all | A little bit | Moderately | Quite a bit | Extremely |
| 10) Blaming yourself or someone else for the stressful experience or what happened after it? | Not at all | A little bit | Moderately | Quite a bit | Extremely |
| 11) Having strong negative feelings such as fear, horror, anger, guilt, or shame? | Not at all | A little bit | Moderately | Quite a bit | Extremely |
| 12) Loss of interest in activities that you used to enjoy? | Not at all | A little bit | Moderately | Quite a bit | Extremely |
| 13) Feeling distant or cut off from other people? | Not at all | A little bit | Moderately | Quite a bit | Extremely |
| 14) Trouble experiencing positive feelings (for example, being unable to feel happiness or have loving feelings for people close to you)? | Not at all | A little bit | Moderately | Quite a bit | Extremely |
| 15) Irritable behaviour, angry outbursts, or acting aggressively? | Not at all | A little bit | Moderately | Quite a bit | Extremely |
| 16) Taking too many risks or doing things that could cause you harm | Not at all | A little bit | Moderately | Quite a bit | Extremely |
| 17) Being “superalert” or watchful or on guard? | Not at all | A little bit | Moderately | Quite a bit | Extremely |
| 18) Feeling jumpy or easily startled? | Not at all | A little bit | Moderately | Quite a bit | Extremely |
| 19) Having difficulty concentrating | Not at all | A little bit | Moderately | Quite a bit | Extremely |
| 20) Trouble falling or staying asleep? | Not at all | A little bit | Moderately | Quite a bit | Extremely |

**Pittsburgh Sleep Quality Index (PSQI)**

The following questions relate to your usual sleep habits during the past month only. Your answers should indicate the most accurate reply for the majority of days and nights in the past month. Please answer all questions.

| Question |
| --- |
| 1) During the past month, what time have you usually gone to bed at night? |
| 2) During the past month, how long (in minutes) has it usually taken you to fall asleep each night? |
| 3) During the past month, what time have you usually gotten up in the morning? |
| 4) During the past month, how many hours of actual sleep did you get at night? (This may be different than the number of hours you spent in bed.) |

| 5) During the past month, how often have you had Not during Less than Once or Three or more trouble sleeping because you… |  |  |  |  |
| --- | --- | --- | --- | --- |
| a. Cannot get to sleep within 30 minutes | Not during the past month | Less than once a week | Once or twice a week | Three or more times a week |
| b. Wake up in the middle of the night or early morning | Not during the past month | Less than once a week | Once or twice a week | Three or more times a week |
| c. Have to get up to use the bathroom | Not during the past month | Less than once a week | Once or twice a week | Three or more times a week |
| d. Cannot breathe comfortably | Not during the past month | Less than once a week | Once or twice a week | Three or more times a week |
| e. Cough or snore loudly | Not during the past month | Less than once a week | Once or twice a week | Three or more times a week |
| f. Feel too cold | Not during the past month | Less than once a week | Once or twice a week | Three or more times a week |
| g. Feel too hot | Not during the past month | Less than once a week | Once or twice a week | Three or more times a week |
| h. Have bad dreams | Not during the past month | Less than once a week | Once or twice a week | Three or more times a week |
| i. Have pain | Not during the past month | Less than once a week | Once or twice a week | Three or more times a week |
| j. Other reason(s), please describe: | Not during the past month | Less than once a week | Once or twice a week | Three or more times a week |
| 6) During the past month, how often have you taken medicine to help you sleep (prescribed or “over the counter”)? | Not during the past month | Less than once a week | Once or twice a week | Three or more times a week |
| 7) During the past month, how often have you had trouble staying awake while driving, eating meals, or engaging in social activity? | Not during the past month | Less than once a week | Once or twice a week | Three or more times a week |
| 8) During the past month, how much of a problem has it been for you to keep up enough enthusiasm to get things done? | No problem at all | Only a very slight problem | Somewhat of a problem | A very big problem |
| 9) During the past month, how would you rate your sleep quality overall? | Very good | Fairly good | Fairly bad | Very bad |
| 10) Do you have a bed partner or room mate? | No bed partner or room mate | Partner/room mate in other room | Partner in same room but not same bed | Partner in same bed |
| 11) If you have a room mate or bed partner, ask him/her how often in the past month you have had: |  |  |  |  |
| a. Loud snoring | Not during the past month | Less than once a week | Once or twice a week | Three or more times a week |
| b. Long pauses between breaths while asleep | Not during the past month | Less than once a week | Once or twice a week | Three or more times a week |
| c. Legs twitching or jerking while you sleep | Not during the past month | Less than once a week | Once or twice a week | Three or more times a week |
| d. Episodes of disorientation or confusion during sleep | Not during the past month | Less than once a week | Once or twice a week | Three or more times a week |
| e. Other restlessness while you sleep, please describe: | Not during the past month | Less than once a week | Once or twice a week | Three or more times a week |

**Socio-demographics**

| Sex assigned at birth | Male |
| --- | --- |
|  | Female |
|  | Other |
|  | Prefer not to answer |
| Gender identity same as sex assigned at birth | Yes |
|  | No |
|  | Prefer not to say |
| How old are you (years)? | [Free text] |
| What is your marital status? | Single |
|  | Married or living with a partner |
|  | Divorced |
|  | Widowed |
|  | Other |
| Wat is your employment status? | Employed: Full-time |
|  | Employed: Part-time |
|  | Self-employed/Freelance |
|  | Not working, looking after the home |
|  | Not working, seeking employment |
|  | Retired |
|  | Other |
| What is your current occupation? | [Free text] |
| What is your ethnicity? | Asian/ Asian British |
|  | Black/ African/ Caribbean/ Black British |
|  | White British |
|  | Mixed/ Multiple ethnic groups |
|  | Other |
| Please indicate your educational attainment. | School until 16 years/ GCSE |
|  | School/ college until 18 years/ A levels |
|  | Further education (e.g., college, vocational training) |
|  | Higher education (undergraduate degree) |
|  | Master’s degree |
|  | Doctoral Degree (Ph.D., M.D., etc.) |
| Have you served in the UK Armed Forces? | Yes |
|  | No |
|  | Currently serving |
| Military branch | Army |
|  | Airforce |
|  | Navy |
|  | Marine |
|  | Corps |
|  | Coast Guard |
| Are you a reservist? | Yes |
|  | No |
| How long did you serve in the armed forces? | [Free text] |
| Which best describes your reason for leaving | Completed term of service |
|  | Better employment prospects in civilian life |
|  | Impact of Service life on family |
|  | Work not exciting or challenging |
|  | Dissatisfaction with pay |
|  | Lack of promotion prospects |
|  | Difficult to plan life outside of work |
|  | Due to deployment |
|  | Pressure on family |
|  | Didn’t want to be aware from home |
|  | My service was terminated |
|  | Health problems |
|  | Pregnancy |
|  | Accomplished everything I wanted |

**UCLA-3**

The next questions are about how you feel about different aspects of your life. For each one, tell me how often you feel that way.

| Question | Score | | |
| --- | --- | --- | --- |
|  | 1 | 2 | 3 |
| 1) First, how often do you feel that you lack companionship:   Hardly ever, some of the time, or often? | Hardly Ever | Some of the time | Often |
| 2) How often do you feel left out:   Hardly ever, some of the time, or often? | Hardly Ever | Some of the time | Often |
| 3) How often do you feel isolated from others?   (Is it hardly ever, some of the time, or often?) | Hardly Ever | Some of the time | Often |

**Short Warwick-Edinburgh Mental-wellbeing scale (WEMWBS-7)**Below are some statements about feelings and thoughts. Please tick the box that best describes your experience of each over the last 2 weeks.

| Question | Score | | | | |
| --- | --- | --- | --- | --- | --- |
|  | 1 | 2 | 3 | 4 | 5 |
| 1) I’ve been feeling optimistic about the future | None of the time | Rarely | Some of the time | Often | All of the time |
| 2) I’ve been feeling useful | None of the time | Rarely | Some of the time | Often | All of the time |
| 3) I’ve been feeling relaxed | None of the time | Rarely | Some of the time | Often | All of the time |
| 4) I’ve been dealing with problems well | None of the time | Rarely | Some of the time | Often | All of the time |
| 5) I’ve been thinking clearly | None of the time | Rarely | Some of the time | Often | All of the time |
| 6) I’ve been feeling close to other people | None of the time | Rarely | Some of the time | Often | All of the time |
| 7) I’ve been able to make up my own mind about things | None of the time | Rarely | Some of the time | Often | All of the time |
